# Correlation Between Blood Glucose Fluctuations and Osteoporosis in Type 2 Diabetes Mellitus

**DOI:** 10.1155/ije/8889420

**Published:** 2025-01-23

**Authors:** Fuhua Chen, Ping Wang, Fang Dai, Qiu Zhang, Ruixue Ying, Liya Ai, Yiqing Chen

**Affiliations:** ^1^Department of Endocrinology, The First Affiliated Hospital of Anhui Medical University, Hefei, Anhui, China; ^2^Department of Endocrinology, The 2nd People's Hospital of Anhui, Hefei, Anhui, China

**Keywords:** blood glucose fluctuations, diabetic osteoporosis, fractures, Type 2 diabetes mellitus

## Abstract

The purpose of this review is to investigate the impacts of blood glucose fluctuations on diabetic osteoporosis, a complication of Type 2 diabetes mellitus (T2DM) that remains poorly understood. We reviewed the current evidence of the relationship between blood glucose fluctuations and diabetic osteoporosis in patients with T2DM. The findings indicate that blood glucose fluctuations may contribute to inhibiting the processes of bone formation and resorption, promoting diabetic osteoporosis and fractures in T2DM. Mechanistic studies, both *in vitro* and *in vivo*, reveal that these effects are largely mediated by oxidative stress, advanced glycation end products, inflammatory mediators, and multiple pathways inducing cell apoptosis or autophagy. Thus, maintaining the long-term stability of blood glucose levels emerges as a target to be pursued in clinical practice in order to safely reduce mean blood glucose and for its direct effects on osteoporosis and fractures in T2DM.

## 1. Introduction

Diabetes mellitus (DM) and osteoporosis (OP) are common chronic metabolic disorders, characterized by high population prevalence, complex etiology, many complications, and serious consequences of complications. In 2021, it is estimated that 537 million people have diabetes, and this number is projected to reach 643 million by 2030, and 783 million by 2045. It is also estimated that over 6.7 million people aged 20–79 will die from diabetes-related causes in 2021 [1]. Meanwhile, the global prevalence of OP stands at 19.7%, and the incidence of osteoporotic fractures, particularly hip fractures, is also on the rise globally. The estimated total annual number of hip fractures nearly doubled from 2018 to 2050 [2, 3]. Therefore, they have grown to be the two major public health problems worldwide. In addition to common chronic complications of diabetes such as diabetic macroangiopathy, diabetic retinopathy, diabetic nephropathy, and diabetic peripheral neuropathy, diabetic OP (DOP) has been receiving growing attention. Studies have shown that diabetic patients have a significantly multiplied risk of OP in comparison to healthy people. Meanwhile, poor blood glucose control can promote fast progression of OP, even causing osteoporotic fractures, posing a continuous risk to the bone health of diabetic patients [4, 5]. Type 2 DM (T2DM) patients make up the vast majority of diabetic populations, hence being a high-risk group for the development of DOP. Due to the occult onset of T2DM and OP, they may have an absence of symptoms in the early stages. Therefore, most patients are only diagnosed when symptoms become evident or fractures occur. As a result, it leads to reduced quality of life, increased mortality, and huge medical expenses, as well as increasing the burden on social healthcare resources. Blood glucose monitoring plays a crucial role in the prevention and treatment of T2DM–related OP. In the past, we focused purely on fasting blood glucose, postprandial blood glucose, and glycated hemoglobin (HbA1c) for blood glucose monitoring. However, in recent years, we have paid more attention to blood glucose fluctuations. A growing number of studies have found that compared to sustained high blood glucose levels, high blood glucose variability (GV) plays a greater role in the occurrence and development of chronic complications of diabetes, such as macrovascular and microvascular complications of diabetes. Possible mechanisms involve the exacerbation of oxidative stress and inflammatory responses; elevated production of advanced glycosylation end products (AGEs) and various inflammatory mediators; and damage of multiple cell types including pancreatic β-cells, vascular endothelial cells, and renal endothelial cells, and inducing their apoptosis [6–8]. Over the past few years, there has been an increasing focus on the relationship between blood glucose fluctuations and DOP and fractures in both basic and clinical research. Therefore, this article reviews the correlation between blood glucose fluctuations and bone turnover markers (BTMs), bone mass density (BMD), fracture risk, and possible mechanisms involved in patients with T2DM.

## 2. Monitoring and Evaluation of Indicators of Blood Glucose Fluctuations

Blood glucose fluctuations, also known as GV, refer to swings in blood glucose levels. The oscillations that occur include short-term blood glucose fluctuations (daily and day-to-day blood glucose fluctuations) and long-term blood glucose fluctuations (i.e., variability of HbA1c) [9]. Healthy individuals typically have a narrow range of blood glucose fluctuations, with blood glucose fluctuating between 3.9 and 7.8 mmol/L for about 96% of the time [10]. However, in diabetes, especially in patients with poor blood glucose control, the range of blood glucose fluctuations far exceeds that of healthy individuals. Previous research showed that the harm of diabetes and its related complications lie in chronic sustained high blood glucose levels. However, emerging evidence has suggested that the harm of high blood GV may outweigh that of sustained high blood glucose [11]. Therefore, in recent years, attention has been paid to monitoring blood glucose fluctuations increasingly.

### 2.1. Monitoring of Blood Glucose Fluctuations

Blood glucose fluctuations' monitoring methods are mainly classified into two categories as follows: continuous glucose monitoring (CGM) and self-monitoring of blood glucose (SMBG). CGM is one of the newest blood glucose measurement technologies applied in clinical practice in recent years. It is mainly used for continuously measuring the changes every 1–5 min in blood glucose levels in individuals with diabetes. It can record blood glucose values for up to 14 days, providing relatively accurate information on glucose fluctuations. Meanwhile, CGM can also guide and optimize treatment decisions for diabetes based on the trends formed by its readings [12]. However, CGM is relatively expensive, operationally complex, and not easily accessible to diabetic patients. On the other hand, SMBG refers to the act of checking capillary blood glucose levels using a blood glucose meter. It can monitor blood glucose at multiple points, such as fasting, pre- and postprandial, and bedtime blood glucose, to identify patterns such as hypoglycemia and postprandial blood glucose fluctuations. SMBG provides retrospective data on the effects of food, activity, lifestyle changes, and medication on blood glucose control, allowing individuals to effectively participate in the management of blood glucose [12, 13]. It is characterized by fast, convenient, relatively inexpensive, and generally accurate but may miss some nighttime blood glucose fluctuations' information. In addition, the variability in blood glucose levels is large. Moreover, some indicators used to assess blood glucose fluctuations are not only complex to calculate but also lack normal reference values.

### 2.2. Evaluation Indicators of Blood Glucose Fluctuations

Currently, commonly used indicators to evaluate blood glucose fluctuations quantitatively are as follows: the coefficient of variance (CV), mean blood glucose (MBG), standard deviation of blood glucose (SDBG), mean amplitude of glycemic excursions (MAGEs), postprandial blood glucose fluctuation amplitude (PPGE), largest amplitude of glycemic excursion (LAGE), mean of daily differences (MODDs), percentage of time (PT), area under the curve (AUC), and low blood glucose index (LBMI). Indicators often applied in CGM include SDBG, MAGE, LAGE, and MODD, while those in SMBG include CV, SDBG, PPGE, and LAGE [8, 14, 15]. Among these Indicators, MAGE is considered as the “gold standard” for reflecting intraday blood glucose fluctuations, calculated as the arithmetic average of either the upward or downward of all glycemic excursions exceeding the threshold (SDBG obtained from all blood glucose concentrations within a 24-h period). the direction of the calculation is determined by the first countable excursion [16]. The absolute MODD using hourly blood sampling was developed as a supplement to MAGE and MBG testing. This approach calculates the mean absolute value of the differences between glucose values on two consecutive days at the same time [17]. Some studies use glycated albumin (GA) to reflect blood glucose fluctuation levels over the past 2-3 weeks or the ratio of GA to HbA1c (GA/HbA1c) to reflect shorter-term blood glucose conditions, with high GA/HbA1c reflecting significant blood glucose fluctuations [18]. In addition, researchers utilize fasting plasma glucose (FPG)–CV, FPG–the average successive variability (ASV), and FPG–variability independent of the mean (VIM) to assess blood glucose fluctuations [19, 20]. Despite many recommended indicators, there is still a lack of a unified and clear gold standard measurement method.

## 3. Clinical Research

### 3.1. The Correlation Between Blood Glucose Fluctuations and BTMs

BTMs are a series of protein or protein derivative biomarkers released into the bloodstream and urine during the process of bone remodeling by osteoblasts or osteoclasts. The markers of bone formation include N-terminal propeptide of Type 1 precollagen (P1NP) and osteocalcin (OC), and those of bone resorption include C-terminal peptide cross-linking of Type I collagen (CTX) and tartrate-resistant acid phosphatase (TRAP). These markers can reflect early bone metabolism status.

Clinical studies have indicated that blood glucose fluctuations are closely correlated with BTMs in patients with T2DM. In a study involving 250 T2DM patients monitored for blood glucose by CGM for three days, Yang et al. [21] found a significant negative correlation between MAGE and BTMs (including P1NP and β-CTX). Similarly, Starup-Linde et al. [22] also found the same results in a study of 100 T2DM patients. Another study monitored 376 T2DM patients with CGM and observed that OC was negatively correlated with GV indicators (including SDBG, MBG, MODD, mean daily risk range [ADDR], and MAGE) [23]. The characteristic of bone metabolism in T2DM–osteoporosis is a state of low bone turnover, causing impaired osteoblast activity and bone repair capacity [24]. The studies above on the correlation between blood glucose fluctuations and BTMs indicate that greater blood glucose fluctuations are associated with lower bone formation and resorption, as well as fragile bone.

### 3.2. The Correlation Between Blood Glucose Fluctuations and BMD

Regarding research on the correlation between T2DM and BMD, it is commonly found that although the risk of fractures is significantly increased in T2DM patients, the association of T2DM with BMD remains inconsistent in the literature. Many authors have noted varied effects. Some studies found that the BMD of T2DM patients was usually normal or even slightly elevated compared to age-matched control groups [25, 26]. However, Jang et al. [27] reported a decrease in BMD among T2DM patients. The studies by Shu and Farr et al. [28, 29] both found that there was no evidence that T2DM produces any change in bone mass. Many factors, such as body mass index (BMI), insulin, estrogen, and leptin will influence the results of BMD [30–32]. The explanation for the increased fracture risk in Type 2 diabetes patients despite the lack of association with BMD is that decreased bone strength is not only attributed to decreased bone density but also to damaged bone quality. Possible mechanisms for that may be associated with reduced bone turnover and impaired osteoblast activity [33, 34]. There have been few clinical studies on the correlation between blood glucose fluctuations and BMD in T2DM. In a cross-sectional study published by Huang et al. [35] in 2022, T2DM patients were divided into groups based on BMD, and the correlation between blood glucose fluctuations and OP was investigated. The results showed that blood glucose fluctuation indicators, including MBG, SDBG, CV, and MAGE, were significantly elevated in the OP group. Multivariate regression analysis' results indicated that MAGE was an independent risk factor for OP. This shows that blood glucose fluctuation indicators, especially MAGE, are closely associated with OP in T2DM patients. In the future, more clinical studies are needed to clarify the correlation between blood glucose fluctuations and BMD in T2DM.

### 3.3. Blood Glucose Fluctuations and Risk of Fractures

Due to low bone turnover, large blood glucose fluctuations may lead to the accumulation of microfractures, ultimately resulting in clinical fractures [22]. Studies have indicated that even in nondiabetic individuals, blood glucose fluctuations may be a new risk factor for osteoporotic fractures [36]. Chiang et al. [20] investigated the relationship between FPG–CV and the risk of hip fractures in elderly T2DM patients. The results showed that patients with FPG–CV higher than 25.4% had an increased risk of hip fractures, indicating that FPG–CV could be used as a predictive factor for hip fractures in elderly T2DM patients. Similarly, the study by Liu et al. [37] found that increased FPG–SD was an independent predictor of osteoporotic fractures in T2DM patients. In addition to FPG variability, the HbA1c variability is also strongly associated with hip fractures in T2DM patients. Lui et al. [38] conducted a correlation analysis on whether the HbA1c–CV can predict the occurrence of hip fractures in T2DM patients. The results showed that HbA1c–CV independently increased the risk of hip fractures in elderly T2DM patients. Another cohort study involving 4,80,539 diabetic patients also found that higher HbA1c–CV, HbA1c–VIM, and HbA1c–average real variability (ARV) were significantly associated with increased incidence and risk of hip fractures [39]. Therefore, in conclusion, high variability in blood glucose can greatly increase the risk of osteoporotic fractures, especially hip fractures, in T2DM patients.

These clinical studies (Table 1) suggest that blood glucose fluctuations may be associated with osteoporotic effects, potentially through mechanisms such as inhibition of bone formation and resorption, reduced bone strength, increased bone fragility, and a higher risk of fractures. However, further prospective studies are needed to establish a causal relationship.

## 4. Mechanistic Research

Clinical studies have confirmed that high blood GV is more harmful to the body than sustained high blood glucose levels, and there are many studies on the mechanisms involved, which may include the following.

### 4.1. Induction of Oxidative Stress Response and Increased Formation of AGEs

**Figure 1:**
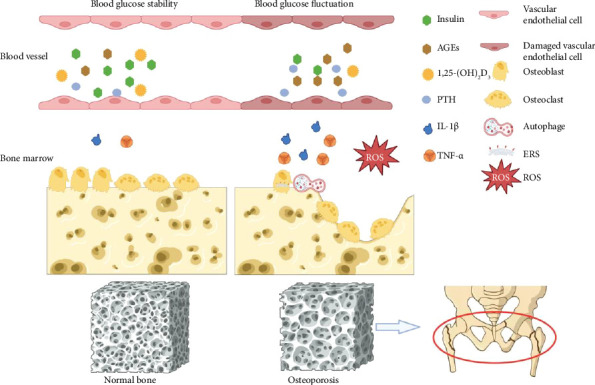
Pathogenesis of diabetic osteoporosis caused by blood glucose fluctuations. Blood glucose fluctuations can lead to oxidative stress, increased levels of advanced glycation end products and inflammatory mediators, reduced insulin secretion, decreased 1,25-(OH)_2_D_3_, elevated PTH levels, and the occurrence of ESR and autophagy. These factors, in turn, inhibit the function of osteoblasts and osteoclasts, leading to osteoporosis and osteoporotic fractures (image created with BioRender.com).

The body's homeostasis is maintained by the balance between the oxidative system (mainly reactive oxygen species [ROS]) and the antioxidant defense system (the main endogenous antioxidants include superoxide dismutase [SOD], catalase, and glutathione peroxidase [GPx]). Excessive production of ROS plays a key role in various physiological and pathological processes [40], such as oxidation of DNA, lipid membranes, and proteins; impaired antioxidant defense systems; activation of downstream pathways associated with oxidative stress; and indirect damage of cells [41, 42]. In both *in vivo* and *in vitro* studies, blood glucose fluctuations have been found to significantly elevate ROS levels compared to chronic hyperglycemia (Figure 1). *In vitro* experiments on human umbilical vein endothelial cells have shown that ROS production was significantly increased in cells cultured with intermittent hyperglycemia compared to constant hyperglycemia, leading to increased apoptosis of human umbilical vein endothelial cells [43, 44]. In *in vivo* experiments, Horvath et al. investigated the effect of blood glucose on oxidative stress in diabetic rats. The rats were divided into a blood glucose stable group, which received long-acting insulin daily, and a blood glucose fluctuating group, which received long-acting insulin every other day. After 14 days, it was found that compared to rats with stable and normal blood glucose levels, rats with blood glucose fluctuations had significantly higher nitrotyrosine levels and endothelial dysfunction [45]. In terms of the influence of blood glucose fluctuations on bone cells, Zhang et al. found that blood glucose fluctuations could lead to a rapid increase in ROS levels and induction of oxidative stress, which could damage osteoblasts, inhibit osteoblast activity and proliferation, and induce osteoblast apoptosis finally [46].

Studies in diabetic patients have also found that overproduction of ROS may be more closely related to blood glucose fluctuations [46–48]. Several experiments have used CGM to investigate the relationship between blood glucose fluctuations and oxidative stress. In a study involving 21 patients with T2DM, MAGE was found to have a strong positive correlation with markers of oxidative stress (24-h urinary excretion rate of 8-iso-PGF2a) [49]. Similarly, in 26 T2DM patients treated with diet and/or metformin, there was a significant association between blood glucose fluctuations and 24-h excretion rate of PGF2a [50]. Ohara et al. found that improvement in blood glucose fluctuations could reduce oxidative stress in patients with T2DM, which further confirms the relationship between blood glucose fluctuations and oxidative stress [51]. However, clinical studies on oxidative stress markers, OP, and bone metabolism in T2DM patients are very limited. In a small-scale study involving postmenopausal T2DM women, a 50% reduction in circulating osteogenic precursors (a type of circulating osteogenic precursors that may enter bone formation sites via blood vessels) was observed, as well as increased expression of oxidative stress marker p66 (Shc) and the antioxidant defense enzyme SOD in these cells [52]. This is consistent with the results of in vitro experiments, indicating increased oxidative stress and decreased osteoblasts in patients with T2DM. Therefore, it is inferred that improving blood glucose fluctuations may also improve oxidative stress levels and osteoblastogenesis, but more clinical studies are still needed to confirm.

In addition, severe oxidative stress reactions cause excessive production of AGEs in the body, which can accumulate in bone tissue along with the bloodstream (Figure 1). This accumulation results in reduced synthesis and increased degradation of Type I collagen, hardening the bone collagen matrix and damaging the toughness and elasticity of bone [53]. Oxidized or glycated nonenzymatic AGEs can bind to a variety of nonenzymatic glycosylated end-product receptors on cell surfaces in the bloodstream and promote the synthesis and secretion of tumor necrosis factor alpha (TNF-α) and interleukin-6 (IL-6), which can inhibit osteoblast differentiation and induce osteoblast apoptosis. Moreover, they can stimulate the transformation of osteoclast precursor cells into osteoclasts and increase osteoclastogenesis, thereby reducing bone formation, increasing bone resorption, and ultimately decreasing bone strength [54–56].

### 4.2. Promoting the Release of Inflammatory Mediators

Inflammation is the primary physiological immune system response and plays a crucial role in the development and progression of diabetic complications. The activation of infiltrating inflammatory cells subsequently activates immune system cells and promotes the production of inflammatory mediators [57]. The release of inflammatory mediators such as cytokines can be mediated by high glucose (HG) or glucose fluctuations and oxidative stress [58]. Compared to sustained hyperglycemia, fluctuating high blood glucose induces a more severe inflammatory response, which is more likely to promote the infiltration of inflammatory cells and mediate the release of various inflammatory mediators, subsequently causing cell and molecular damage (Figure 1). Li et al. [59] made experimental peri-implantitis models in diabetic db/db mice. Glycemic control or fluctuation was managed by constant or interrupted oral administration of rosiglitazone to these mice. They found that compared to mice with sustained high blood glucose, diabetic mice with fluctuating blood glucose exhibited greater bone loss, inflammatory cell infiltration, and osteoclastogenesis of peri-implant tissues. This may be related to the transition of the peri-implant microbiome toward dysbiosis and activation of the TLR2/4-IRAK1-TRAF6 signaling pathway. Moreover, compared to sustained high blood glucose, it was found that blood glucose fluctuations led to increased expression of inflammatory factors such as interleukin-1β (IL-1β) and TNF-α in gingival tissues surrounding implants. In addition, ROS can also aggravate cellular inflammation and apoptosis by activating the profibrotic signaling pathway mediated by the transforming growth factor-β (TGF-β) [60], which could promote osteoblast apoptosis and reduce bone formation. Therefore, there is a complex relationship between the inflammatory response and oxidative stress response, which can interact and amplify each other.

### 4.3. Multiple Pathways Inducing Cell Apoptosis or Autophagy

#### 4.3.1. Osteoblasts

Osteoblasts play an important role in bone formation and bone mass maintenance. In addition to factors such as oxidative stress and release of inflammatory mediators that can lead to osteoblast apoptosis, there are other pathways which can inhibit osteoblast activity or proliferation (Figure 1). Zhang et al. [46] studied MC3T3-E1 osteoblasts and found that HG and glucose fluctuations inhibited the proliferation of MC3T3-E1 osteoblast and induced cell apoptosis and autophagy, with fluctuating high blood glucose being more obvious. Meanwhile, they also found that osteoblast apoptosis increased after the use of autophagy inhibitors, suggesting that autophagy induced by HG and glucose fluctuations may protect MC3T3-E1 osteoblasts from apoptosis, and this autophagic effect may be related to the AKT signaling pathway [61]. Shuai et al. found that the activity, differentiation, and mineralization of MC3T3-E1 cells in the fluctuating HG (VHG) group were lower than those in the sustained HG group. At the same time, autophagy was significantly increased in the VHG group, and it was found that endoplasmic reticulum stress was also significantly enhanced in the VHG group. In conclusion, glucose fluctuations can inhibit osteoblast proliferation and differentiation by stimulating ERS [62].

#### 4.3.2. Endothelial Cells

The relationship between the vascular system and the skeleton is inextricably linked. Cells of the vascular system, such as vascular endothelial cells, participate in the development, reconstruction, and nourishment of bones, promoting bone growth and healing after injury [63]. If the vascular system is damaged, bone homeostasis can be impaired (Figure 1) [64]. In T2DM, blood glucose fluctuations were found to cause an impaired function or apoptosis of vascular endothelial cells through the protein kinase C (PKC) signaling pathway [43]. Endothelial cell apoptosis then leads to reduced signaling for the recruitment and differentiation of osteoprogenitor cells. This diminished signaling inhibits the recovery of bone formation at cortical remodeling sites, resulting in impairments in bone material properties and increased cortical porosity [65]. In the clinical experiment, Samakkarnthai et al. found an increase in cortical porosity at the distal tibia in 171 T2DM patients with microvascular complications following high-resolution peripheral quantitative computed tomography (HR-pQCT) scanning of the tibia, which potentially contributes to increased fracture risk in T2DM patients [66].

#### 4.3.3. Pancreatic β-Cells

Insulin has been shown to be an osteogenic hormone *in vitro* and *in vivo*. *In vivo* experiments have demonstrated that insulin could prevent or reverse bone strength and biomechanical integrity of diabetic mice. Meanwhile, *in vitro* experiments have also confirmed that the combination of primary osteoblasts and osteoblast-like cells with insulin could elevate the cell proliferation rate, enhance collagen synthesis, and increase the secretion of bone formation markers [67, 68]. In addition, in patients with T2DM, greater glycemic variability corresponds to poorer pancreatic islet β cell function (Figure 1). Studies have found that MAGE was negatively correlated with the function of islet β-cells in CGM, suggesting that glycemic variability parameters can serve as indirect indicators to evaluate the function of islet β-cells [69, 70]. The insulin-dependent process of glucose uptake and utilization involves the protein kinase B (PKB/AKT) signaling pathway [71]. Shao et al. [72] found that fluctuating HG levels could exacerbate damage to rat islet cell tumor cells (tINS-1) by inhibiting insulin signaling. Moreover, under conditions of blood glucose fluctuations, the expression of phosphatase and tensin homolog (PTEN) increases. The lipid phosphatase activity of PTEN could dephosphorylate phosphatidylinositol 3-phosphate (PIP3) and negatively regulate the phosphatidylinositol 3-kinase (PI3K)/AKT pathway. Consequently, decreased levels of phosphorylated AKT mediate damage and apoptosis of pancreatic islet β-cells, resulting in reduced insulin secretion and decreased stimulation of osteoblastogenesis. Therefore, blood glucose fluctuations can cause apoptosis or impaired function of pancreatic β-cells, thereby affecting bone formation and leading to OP.

#### 4.3.4. Renal Endothelial Cells

Arnson, Amital, and Shoenfeld [73] also mentioned in their study that blood glucose fluctuations can damage renal endothelial cells, resulting in decreased activity of the key enzyme (1-α hydroxylase) involved in the synthesis of active vitamin D and then reduced synthesis of 1,25-dihydroxyvitamin D. Consequently, it leads to reduced calcium absorption from the intestines. Hypocalcemia, vitamin D deficiency, and excessive secretion of parathyroid hormone (PTH) caused by hypocalcemia contribute to decreased raw materials for bone formation, increased bone resorption, and raised risk of OP and osteoporotic fractures (Figure 1).

## 5. Conclusions and Future Prospects

In summary, there are many studies supporting the association of blood glucose fluctuations with the development and progression of DOP and fractures in T2DM patients. The results of experimental and clinical research demonstrate that blood glucose fluctuations may contribute to inhibiting the processes of bone formation and resorption and are the risk factors of DOP and fractures. Therefore, in the management of T2DM patients, more attention should be paid to controlling the absolute blood glucose levels and fluctuations to minimize glycemic swings. Examinations should be advised to prevent DOP and fractures early, including regular bone density examinations and metabolism indicators tests when necessary. T2DM patients need to increase exposure to sunlight while taking precautions against falls. For the patients who have already been diagnosed with DOP or fractures, proper glycemic management and reduction of blood glucose fluctuations are crucial, along with prompt initiation of treatment with antiosteoporotic medications.

Although many studies have suggested a relationship between blood glucose fluctuations and DOP and fractures in T2DM patients, significant gaps remain in understanding the underlying mechanisms and the long-term clinical implications of these fluctuations on bone metabolism. The existing body of literature largely consists of cross-sectional and retrospective studies, which, while informative, cannot establish causal relationships or fully elucidate the biological mechanisms involved.

One key limitation of current studies is the lack of longitudinal research that tracks the effects of blood glucose fluctuations over time. Longitudinal studies could provide valuable insights into how prolonged fluctuations in blood glucose levels contribute to the development of OP and increased fracture risk in T2DM patients. By following patients over extended periods, such studies could better capture the chronic impacts of GV on bone turnover, BMD, microstructure, and fractures, thus providing more robust evidence of causality.

Furthermore, interventional trials are needed to assess the impact of interventions aimed at stabilizing blood glucose levels on DOP. For instance, studies that evaluate the effects of glucose-lowering therapies, dietary modifications, or lifestyle interventions on reducing blood glucose fluctuations could help determine whether managing GV can effectively reduce bone fragility and fracture risk in T2DM patients. Such trials could also provide insights into the optimal targets for blood glucose control in relation to bone health.

In addition, research on the mechanisms through which blood glucose fluctuations impact DOP is still limited. While oxidative stress, AGEs, and inflammation have been proposed as potential mediators, the specific cellular and molecular pathways remain poorly understood. Further investigation into how blood GV influences osteoblast and osteoclast activity, as well as its effect on bone matrix proteins, is essential. This would help clarify the biological processes that link blood glucose fluctuations to the disruption of bone metabolism in DOP.

In summary, while the current literature suggests a link between blood glucose fluctuations and DOP in T2DM, there is a pressing need for more robust and comprehensive studies. Longitudinal studies, interventional trials, and mechanistic research are essential to uncover the underlying mechanisms and to provide evidence-based strategies for managing bone health in diabetic populations.

## Figures and Tables

**Table 1:** Summary of clinical studies investing in blood glucose fluctuations and their association with bone health in Type 2 diabetes mellitus.

Study, year	Clinical research types	Patient numbers	Blood glucose fluctuations measure	Osteoporosis-related indicators	Results
Yang et al. 2023 [21]	Cross-sectional study	250	MAGE	P1NP and β-CTX	MAGE was negatively correlated with P1NP and β-CTX.
Starup-Linde et al. 2021 [22]	Cross-sectional study	100	MAGE	S-CTX, s-P1NP, and s-sclerostin	S-CTX was significantly negatively associated with MAGE.
Liu et al. 2022 [23]	Cross-sectional study	376	SD, MBG, MODD, ADDR, and MAGE	Osteocalcin	There was a negative correlation between osteocalcin and GV indicators (*P* value of MAGE > 0.05).
Huang et al. 2022 [35]	Cross-sectional study	362	24-h MBG, SDBG, CV, and MAGE,	T-scores	T2DM patients with osteoporosis showed a higher 24-h MBG, SDBG, CV, and MAGE. MAGE independently contributes to osteoporosis
Chiang et al. 2016 [20]	Retrospective cohort study	26,501	FPG–CV	Hip fracture	T2DM patients categorized as FPG–CV greater than 25.4% showed an increased risk of hip fractures.
Liu et al. 2023 [37]	Prospective cohort study	57,295	FPG–SD	Osteoporotic fracture	Increased FPG variability was associated with a greater risk of osteoporotic fractures in people with diabetes than in those without diabetes
Lui et al. 2020 [38]	Prospective cohort study	83,282	HbA1c–SD, HbA1c–AdjSD, and HbA1c–CV	Hip fractures	HbA1c–SD, HbA1c–AdjSD, and HbA1c–CV were significant independent predictors of incident hip fractures.
Lee et al. 2022 [39]	Prospective cohort study	480,539	VIM, CV, and ARV of blood glucose	Hip fractures	High glucose VIM, CV, and ARV were associated with a high risk of hip fracture in diabetic patients.

## Data Availability

Data sharing is not applicable to this article as no new data were created or analyzed in this study.
